# Dairy goat sulfadoxine depletion trial in milk and diagnostic accuracy of the Charm Rapid One Step Assay (ROSA) SULF test

**DOI:** 10.3168/jdsc.2024-0559

**Published:** 2024-06-28

**Authors:** Miranda Hawley, Joe Smith, Kaitlyn Lawson, Jocelyn Jansen, Rex Crawford, Afolakemi Adeniji, Cathy Bauman

**Affiliations:** 1 Ontario Veterinary College, University of Guelph, Guelph, ON, Canada N1G 2W1; 2 SMART Pharmacology, Iowa State University, Ames, IA 50011; 3 Ontario Ministry of Agriculture, Food and Rural Affairs, Elora, ON, Canada, N1G 2W1; 4 Dufferin Veterinary Services, Amaranth, ON, Canada, L9W 0N4

## Abstract

In Canada, currently no antibiotics are approved for use in lactating dairy goats. Trimethoprim sulfadoxine is indicated for the treatment of bacterial pneumonia, enteric and septicemic colibacillosis and salmonellosis, infectious pododermatitis, and septicemias. The aim of this study was to determine the trimethoprim sulfadoxine withdrawal time and evaluate the test accuracy of the Charm Rapid One Step Assay (ROSA) SULF test (Charm Sciences Inc.) at the individual goat level. The study was conducted on 20 visibly healthy Ontario dairy goats. They received trimethoprim sulfadoxine at a dose of 16 mg/kg i.m. once a day for 5 d as the commercially available preparation trimethoprim sulfadoxine (Borgal, Merck Animal Health). Residue levels were measured using liquid chromatography-tandem mass spectrometry and the Charm ROSA SULF. The withdrawal time was calculated using safe concentration linear regression. The determined milk withdrawal time was 60 h.

## Highlights

• Trimethoprim sulfadoxine was injected intramuscularly (3 mL/kg) into lactating goats.
• Milk samples were collected: baseline, every 12 hours for 3 days, then every 24 hours.
• Samples tested for residues by liquid chromatography with tandem mass spectrometry.
• Milk withdrawal time for sulfadoxine in goat milk is 60 hours postinjection.

## Graphical Abstract

**Figure:**
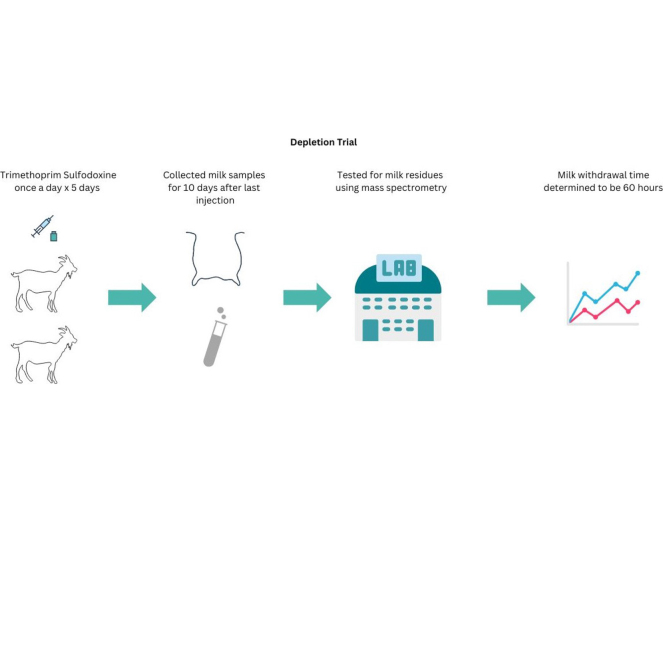
**Summary:** Depletion trials help determine withdrawal times for medications without regulatory approval for minor species. Trimethoprim sulfadoxine was administered to 20 goats once a day for 5 days. Serial milk samples were collected and tested for sulfadoxine residue for 10 days after the last injection. The withdrawal time was determined to be 60 hours after the last injection given based on mass spectrometry testing and the safe concentration from linear regression method using WTM 1.4 software.

In North America, no antibiotics and few veterinary drugs are approved for use in lactating goats. Consequently, antibiotics that are used in goats are used in an extralabel manner (Government of Canada, 2014). As part of the drug approval process, pharmaceutical companies conduct depletion trials to accurately calculate a withdrawal period for the drug of concern. Therefore, in the absence of drug approvals and species-specific withdrawal times for lactating goats, one of the main concerns of extralabel drug use (**ELDU**) is the potential for drug residues in milk to occur. These residues are a potential health risk to human consumers due to the potential for allergic reactions to even low levels of antibiotic residues (e.g., β-lactams and sulfonamides) and they may be carcinogenic or teratogenic in some circumstances (Gavage et al., 2021). Antibiotic residues also have the potential to promote antimicrobial resistance (Baynes et al., 2016).

The regulation of antibiotic use is overseen by governmental authorities in each country (Veterinary Drug Directorate, Health Canada; Food and Drug Administration [**FDA**], United States). These authorities apply country-specific regulations established under the Food and Drugs Act (Canada) and the Food, Drug and Cosmetic Act as well as the Animal Medicinal Drug Use Clarification Act (United States). Both countries allow ELDU within the confines of a veterinary-patient-client relationship. The prescribing veterinarian must provide in writing the “dosage regimen, route and frequency of administration, duration of treatment, and withdrawal intervals to avoid a risk to food safety” (CVMA, 2021). Consequently, veterinarians rely on organizations such as the Canadian global Food Animal Residue Avoidance Databank (**CgFARAD**), and the US version FARAD, to provide accurate withdrawal information. In the absence of direct depletion trial data, they collect, organize, analyze, and share residue avoidance information. Frequently with minor species little to no information is available for these organizations to make withdrawal time recommendations, and therefore there is a need to conduct depletion trials either by government, industry, or other institutions such as academia.

Depletion trials for small ruminants must be conducted in each individual species of interest. Data often cannot be extrapolated from animals similar in size such as sheep to goats, because they all differ physiologically in how they metabolize drugs (e.g., sheep lack the enzyme to metabolize copper whereas goats do not). For the results to be widely accepted, depletion trials are structured on the guidelines of the Veterinary International Conference on Harmonization, an international organization that standardizes the process to obtain veterinary drug approval (VICH, 2015).

Based on regulations in VICH guideline no. 48: Marker Residue Depletion Studies to Establish Product Withdrawal Periods established for milk depletion trials, at least 5 does from each of the 3 stages of lactation: early (<90 d), mid (90–180 d), and late (>180 d), must be selected for a minimum total of 20 lactating animals. Regulatory residue levels are typically measured using liquid chromatography-tandem mass spectrometry (**LC-MS/MS**), an analytic technique that is a hypersensitive method for testing food and water (Rappold, 2022) but is labor intensive, time consuming, and expensive. In the wider dairy industry, processors routinely screen bulk tank milk for drug residues (e.g., β-lactams, tetracyclines) using high-throughput assays such as the Charm ROSA systems (Charm Sciences Inc., St. Lawrence, MA). These tests have previously been validated against LC-MS/MS and are approved for use on raw comingled milk for bovine, sheep, goat, and buffalo milk (Charm Sciences Inc., 2022). Little is known about the performance of these assays at the individual animal level. If they demonstrated sufficient sensitivity and specificity, they could be a more reliable test than some of the farm-level currently available residue tests and could be used in future research projects.

Depletion trials for commonly used antibiotics should be prioritized. Trimethoprim-sulfadoxine is a drug often used in the small ruminant industry (Clark, 2013) due to its low cost and wide efficacy for common bacterial infections. In the United States, extralabel use of most sulfonamides in lactating dairy cattle >20 mo is prohibited due to the concern arising from detected residues in milk (Davis et al., 2009). Currently there is only one sulfonamide (sulfadoxine) approved for ELDU in lactating dairy cattle (530.41; AMDUCA, 1994). In lactating dairy goats and sheep, ELDU of sulfonamides is not prohibited, but not encouraged for the same reason (Davis et al., 2009). To assist in the prevention of residues, the objective of this study is to describe the depletion of sulfadoxine in goat milk, determine a recommended withdrawal time, and investigate the use of the Charm ROSA test on individual goat milk samples.

The farm that participated in this study was a privately owned Ontario-licensed, mid-sized (n = 300 lactating does) commercial dairy goat farm located in Southwestern Ontario that milked their animals twice a day. The herd consisted of purebred Alpines, Saanens, or crosses of the 2 breeds. Twenty-four does in total (4 negative controls) were randomly selected for the trial using random number sets (generated from random.org) as they entered the milking parlor (24 goats per side). Four does, from every group of 48 that entered, were tentatively selected for participation in the study. The inclusion criteria for this study were (1) a lactating doe at least 10 d postpartum, (2) 2 functional udder halves, and (3) had not been treated with any veterinary drugs within the last 30 d. Each doe was subsequently checked for signs of clinical mastitis by visually appraising the udder for redness and swelling and checking the first streams of milk for blood, milk clots, or gray-watery milk, and the goat was excluded from the study if one or more signs were present. Four negative control goats were also randomly selected from the herd to not receive any antibiotic treatments.

All goats meeting the inclusion and exclusion criteria were identified with a red crayon marker, had their ear tag or neck chain numbers recorded, and a baseline milk sample (∼50 mL) was taken, which was a composite sample from both teats into sterile plastic vials (Fisher Scientific, Waltham, MA). Following collection of the baseline samples, the does were hand-milked out into a pail and the milk was discarded. As each enrolled goat left the parlor they were weighed using a digital Marweld scale (Marweld, Millbank, ON, Canada). The weights ranged from 43 to 97.5 kg.

Postweighing, all animals received their first injection of Borgal (200 mg of sulfadoxine and 40 mg of trimethoprim per mL, Merck Animal Health, Kirkland, QC, Canada) intramuscularly in the right side of the neck, using a dosage of 16 mg/kg of combined sulfadoxine and trimethoprim (3 mL per 45 kg; label directions for cattle and pigs) and rounded up to the nearest 0.5 mL. If the dose being given was larger than 5 mL, it was divided in half and given in 2 injection sites on the same side of the neck (Clark, 2013) to reduce the risk of prolonging the absorption phase of the drug. The animals were then segregated in a separate pen for the remainder of the study and continued to be milked twice a day after the main herd had left the parlor (∼0800 and 1900 h) and the milk was discarded. Trimethoprim-sulfadoxine injections continued to be administered every 24 h immediately after milking for the following 4 d, resulting in a total of 5 injections. The site of injection alternated between the right and left sides of the neck each day. All goats were monitored for any adverse effects. The does received the injections while restrained by headgates and a technician in the milking parlor.

Twelve hours following the final antibiotic injection, milk sampling commenced. Three 50-mL composite milk samples were taken from all 24 does at 12, 24, 36, 48, 60, 72, 96, 120, 144, 168, 192, 216, and 240 h postinjection. Before sampling, any physical debris was cleaned from each teat using a wipe (Huggies Natural Care Unscented Baby Wipes), and then the first 3 to 5 streams of milk from the teat were discarded. Each 50-mL sample was labeled with the animal ear tag number and placed in a Styrofoam cooler containing ice. Postsampling, the does were then milked using the farm's automatic milking system and the milk was discarded. The milk from the final day of sampling (d 10) was submitted fresh to the Agriculture and Food Laboratory (**AFL**) for immediate LC-MS/MS testing to ensure no sufadoxine-d3 residues were detectable before re-introducing the milk from the study animals back into the bulk tank.

Milk samples for SCC measurement (Foss Analytics, MN) and Charm ROSA testing were transferred to a refrigerator (4°C–6°C), whereas samples for culture and LC-MS/MS testing were transferred to a −20°C freezer, all within an hour of collection. Frozen samples were then transferred to the relevant laboratory and stored at −80°C every 72 h for future batch analysis. The 3 vials from each animal were distributed for testing as follows: (1) sulfadoxine-d3 residues using the liquid chromatography-tandem mass spectrometry (**LC-MS/MS**) method at the AFL (University of Guelph), (2) all sulfonamide drug residues using the Charm ROSA SULF test (Charm Sciences Inc., Lawrence, MA) according to the manufacturer's instructions at AFL, and (3) total aerobic count and aerobic culture at the Animal Health Laboratory (University of Guelph).

Sample preparation and LC-MS/MS were performed according to an established proprietary AFL protocol based on the following method (Gaugain-Juhel et al., 2009) developed for the identification of sulfonamides in goat milk and extended to include the identification of sulfadoxine. In summary, before LC-MS/MS was performed, the sulfadoxine in the thawed raw goat milk was extracted using 8 mL of acetonitrile. After centrifugation at 7,000 × *g* for 5 min at room temperature, the supernatant was evaporated at 45°C to approximately 50 µL of extract. This volume was then reconstituted with 90:10 (vol/vol) 0.1% formic acid in water: 0.1% formic acid in methanol. The extract was then filtered using a 0.2-µm polytetrafluoroethylene syringe filter and analyzed by LC-MS/MS. Chromatography was carried out using an Agilent Poroshell (120 EC-C18 2.7 μm, 2.1 × 50 mm) placed in a column oven (50°C). The mobile phases were A: 0.1% formic acid H_2_O and B: 0.1% formic acid in methanol. Injection volumes of 1.0 μL of sulfadoxine-d3 (Vetranal, Sigma) were used as an internal standard with a limit of detection (**LOD**) of 0.80 ppb and a limit of quantification (**LOQ**) of 2.67 ppb. The sulfadoxine levels were reported in parts per billion where possible (≥2.67), <minimum quantifiable limit (**MQL**; 0.8 to <2.67), or not detected (**ND**; <0.8).

The Charm ROSA is an immunoreceptor assay utilizing a ROSA. Sulfonamide drugs interact with colored beads in the lateral flow test strip (Charm SULF test for raw commingled cow, goat, and sheep milk), which in turn is read by the Charm EZ ROSA Reader (reads the color intensity in the test and control lines). According to Charm, the level of detection of this test strip for sulfadoxine is 18 ppb (Charm Sciences Inc., 2022). The test uses 300 µL of the sample milk and 300 µL of SULF-MRL dilution buffer (Charm Sciences) is added to the milk. The test strip is incubated for 8 min in the ROSA incubator before being inserted into the EZ reader for reading.

All data were entered into Excel (Microsoft 365) and exported to R/RStudio (R Core Team, 2017). Before generating numerical summary statistics, all variables were checked for normality using histograms and the Shapiro-Wilk test (cut-off of *P* < 0.05). Means and medians were calculated and compared using *t*-tests or the Wilcoxon rank sum test depending on the tests for normality (histogram and Shapiro-Wilk test). Test sensitivity and specificity were generated using LC-MS/MS as the reference test (LOD: 2.67 ppb) and utilizing the package epiR.

All sulfadoxine pharmacokinetic parameters were determined using Phoenix Win-Nonlin 8.0, Certara Inc., Princeton, NJ). Noncompartmental analysis was performed using extravascular dosing, uniform weighting, unconditional below quantifiable limit substitution (MQL and ND = 0), the sparse data option, and the linear log trapezoidal calculation method. The following parameters were subsequently estimated and summarized by their mean values: (1) the peak sulfadoxine residue concentration in milk (C_max_ = 197.6 ppb), (2) the time needed to achieve this peak concentration (T_max_ = 12 h), (3) the standard error for C_max_ (37.3), (4) the peak last concentration of sulfadoxine (C_last_ = 1.54 ppb), (5) the time at C_last_ (T_last_ = 36 h), (6) mean residence time − last (14.1 h), (7) area under the curve − last (2,152.7), (8) area under the curve − all (2,162.0), and (9) area under “m” curve (30,428.8).

Milk withdrawal intervals for sulfadoxine were calculated using European Medicines Agency (**EMA**)'s WTM 1.4 software as previously described for flunixin in goat milk (Smith et al., 2020). This software program is an updated computerized version of a harmonized approach for the calculation of withdrawal periods for milk throughout the European Union. The WTM 1.4 program has been adopted by the Committee for Veterinary Medicine Products of EMA. Several calculation method options are available in this program, including the safe concentration from linear regression (**SCLR**) method and the time-to-safe-concentration (**TTSC**) method. The TTSC method is not applicable to the present data because sulfadoxine has a zero tolerance in goat milk; thus, the tolerance is operationally equivalent to the LOD of the marker residue sulfadoxine-d3 0.8 ppb in milk, which is lower than all the quantifiable concentrations. Therefore, the SCLR calculation method was used to analyze the present concentration data in goat milk. The LOQ of sulfadoxine was 2.67 ppb. The average coefficient of variability of the assay was 2.84%.

Of the 24 does enrolled in the project, LC-MS/MS and Charm ROSA test results were available for all 24 animals at each of the 13 sampling times (Table 1). Total aerobic bacterial counts and culture results were available for baseline, 24 h and 168 h after final injection.

**Table 1:** Demographics of 24 lactating does sampled in a sulfadoxine-d3 (ppb) milk depletion trial conducted in southern Ontario

Stage of lactation	Mean weight, kg (median)	Mean baseline aerobic count, cells/mL (median)	Mean 24-h postinjection aerobic count, cells/mL (median)	Mean 168-h postinjection aerobic count, cells/mL (median)	Mean baseline SCC × 1,000/mL (median)
Early, n = 6	67.1	7,130	5,000	13,300	2,123
(<90 d)	(60.0)	(2,300)	(1,050)	(1,850)	(965)
Mid, n = 9	61.4	6,240	3,650	22,500	654
(90–180 d)	(60.5)	(200)	(1,200)	(600)	(1,266)
Late, n = 9	67.1	4,000	6,700	989	2,610
(>180 d)	(67.5)	(900)	(900)	(500)	(480)
Total	65.0	5,696	5,222	12,117	1,738
	(63.8)	(900)	(800)	(650)	(721,000)

None of the milk samples taken at baseline were positive on LC-MS/MS for sulfadoxine and none of the 4 negative control goats ever tested positive. At 12 h after the last antibiotic injection, all 20 does that had been injected had positive LC-MS/MS concentrations; the sulfadoxine levels at this time point ranged from 16 to 580 ppb with a mean of 197.6 (95% CI: −151.2–546.3) and a median of 145.0. At 60 h after the last injection no detectable sulfadoxine residues were detectable using LC-MS/MS (Figure 1). Based on all samples with a quantifiable LC-MS/MS residue, goats in late lactation had an overall median sulfadoxine level (21.5 ppb) lower than the goats in early (40.4 ppb) and mid lactation (32.2 ppb; *P* = 0.003). This association is contrary to what would be expected as milk yield is at its lowest volume in late lactation. Residue levels did not correlate with the animal's weight (rho = −0.1453; *P* = 0.5410).

**Figure 1:**
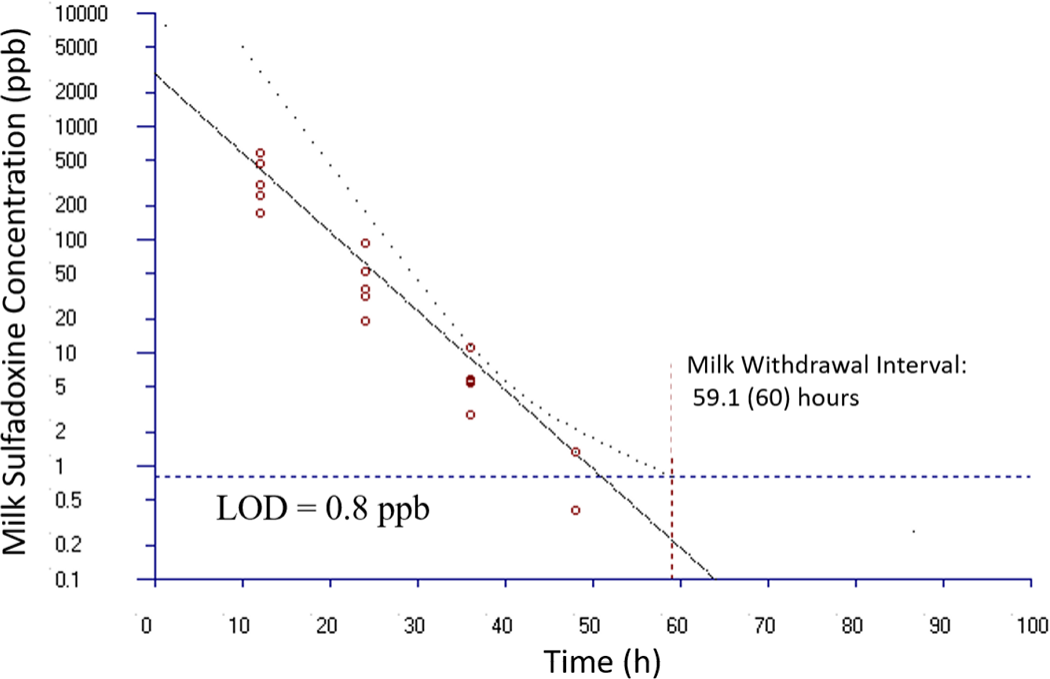
Estimated milk withdrawal interval for sulfadoxine d-3 in lactating goats after 5 intramuscular injections at 16 mg/kg of sulfadoxine. The calculation was based on the milk data of sulfadoxine-d3 (i.e., the marker residue of sulfadoxine in milk) using the European Medicines Agency's WTM 1.4 software (i.e., the EMA method).

The milk withdrawal interval, as calculated by the EMA's WTM 1.4 software SCLR method, was 59.1 h (Figure 1). Not all animals fit the criteria for the SCLR model (at least 3 samples with measurable residues) and were not incorporated into the final withdrawal interval calculation. These animals did not contribute data due to their rapid clearance of sulfadoxine and therefore the proposed withdrawal time is inherently more conservative. Because these data were collected from animals being milked every 12 h, the realistic withdraw interval should reflect that all milk collected up to 60 h is discarded as milk accumulated between the 48- and 60-h milking could still have trace sulfadoxine concentrations present. This recommendation from the model aligns with the observed milk sulfadoxine concentrations in the does in this study, as no animals had detectable concentrations in milk collected 60 h after final administration, and 4/20 treated animals had detectable, but not quantifiable, concentrations at 48 h after administration.

The current label recommendation for the sulfadoxine milk withholding time in Canada for cattle is 96 h. A goat drug withdrawal time that is shorter than the clearance time for other species is not unusual. There are multiple examples of studies where goats have metabolized or cleared drugs more rapidly than sheep or cattle: meloxicam (Shukla et al., 2007), sulfamethazine (Elsheikh et al., 1991), and oxytetracycline (Attaie et al., 2015), but this is not always the case. There are some drugs (e.g., ivermectin) that take the same length of time or longer to clear which may be related to the lack of drug metabolism involved (i.e., drug is excreted unchanged), the extensive protein binding that occurs, or both (González Canga et al., 2009).

The withdrawal time estimated in this study is based on the VICH guidelines, which stipulate that the drugs be administered and tested in healthy animals. Contrary to this, most antibiotics in veterinary practice are administered to animals that are medically compromised in some capacity and likely not clearing the drug as efficiently. Factors such as dehydration, impaired liver metabolism, and reduced kidney excretion can increase the time for drugs to clear. Conditions such as mastitis can also cause some drugs to accumulate in that compartment (e.g., apramycin and ketoprofen in cow milk) at levels up to 10 times higher than would be found in animals without that condition (Beyene, 2016). The SCLR method of calculating the withdrawal time determines the 95% tolerance limits for a given drug, which means that 95% of the drug residue concentrations will fall below the level of detection by 59.1 h with 95% confidence. The risk of milk residues can never be reduced to zero as even the most stringent recommendations of milk withhold are not absolute in their guarantee of residue elimination; however, with common sense a producer and veterinarian can identify the animals and situations that may be higher risk. The other element of risk also relates to the number of animals treated with this medication relative to the number of animals milking in the herd.

While the US FDA uses 99% tolerance limits, the European Union determined that using a sample size of 19 is advisable to achieve a 95% confidence level with an empirical basis (EMEA, 2000). An advantage of the SCLR system is that the data can be analyzed in the absence of replicate test results for the same sample (which occurred in this study), which is required by the FDA method of analysis. Unfortunately, running samples in triplicate doubles the amount of time spent testing the samples (written communication, AFL) and triples the cost of testing.

The median total aerobic bacterial counts in the milk samples did not vary between baseline (900 cfu/mL), 24 h postinjection (800 cfu/mL), and 168 h postinjection (650 cfu/mL; *P* = 0.1681). Approximately 21% of samples did not culture a pathogen at baseline (n = 5). Of those goats with a positive culture, the most common pathogens grown at baseline were (1) *Staphylococcus epidermis* (25%; n = 6), (2) *Staphylococcus caprae* (21%; n = 5), and (3) *Corynebacterium bovis* (12.5%; n = 3). There was no correlation between the LC-MS/MS residue level at 12 h postinjection and (1) the baseline milk aerobic bacterial count (rho = −0.3594; *P* = 0.1307), (2) the 24-h aerobic count (rho = −0.2774; *P* = 0.2362), (3) the 168-h aerobic count (rho = −0.2582; *P* = 0.2717), or (4) the difference in aerobic count between baseline and the 24-h count (rho = 0.1407; *P* = 0.5657). There was fair to moderate correlation between the residue level at 12 h postinjection and SCC (rho = 0.5960; *P* = 0.0071). In drug clearance studies in cows, it has been observed that clearance of antibiotics can either be prolonged in animals with mastitis or there is no difference (Gehring and Smith, 2006). Reasons for prolongation could be increased vascular permeability, deeper penetration into udder tissue, decreased milk production due to the infection, or a combination of these (Gehring and Smith, 2006). No information on milk yield was obtained in this study, but this may be a parameter that should be measured in future depletion studies.

At 60 h after the last sulfadoxine injection, all of the does were also negative on the Charm ROSA test. During the 24- to 48-h sampling period, there were only 3 discordant results when the Charm test was compared with LC-MS/MS results. At 36 h postinjection, goat #147 (mid lactation) was negative on the LC-MS/MS (designated as <MQL; 0.8 to <2.67) and positive on the Charm ROSA and goat #721 (late lactation) was positive on LC-MS/MS (5.6 ppb) and negative on the Charm ROSA. At 48 h, goat #14 (early lactation) was negative on LC-MS/MS (but listed as <MQL) and positive on the Charm ROSA. The lowest test sensitivity for the ROSA test occurred at 36 h (60%; Table 2), but this statistic was based on missing the detection of one positive test that was well below the level of detection that was previously published for this test (18 ppb; Charm Sciences). The test was otherwise able to correctly classify 15 tests with residue levels between 2.67 and 18 ppb. Overall, it otherwise appears to be a relatively sensitive and specific test to use as an alternative to LC-MS/MS for detecting residues in individual goats.

**Table 2:** Test accuracy of the Charm ROSA SULF test for sulfonamide residues, evaluated on individual dairy goat milk samples at 5 sampling time points postinjection with intramuscular trimethoprim-sulfadoxine and using liquid chromatography-tandem mass spectrometry (LC-MS/MS) as the reference test

Hours postinjection	Number of samples with detectable levels on LC-MS/MS	Number positive on Charm ROSA SULF test	Sensitivity (95% CI)	Specificity (95% CI)
12	20	20	1.00 (0.83–1.00)	1.00 (0.40–1.00)
24	18	18	1.00 (0.81–1.00)	1.00 (0.54–1.00)
36	5	4	0.6 (0.15–0.95)	0.95 (0.74–1.00)
48	0	1	NA^1^	0.96 (0.79–1.00)

1 NA = not applicable as no LC-MS/MS samples tested positive at 48 h.
